# Heat-not-burn tobacco, electronic cigarettes, and combustible cigarette use among Japanese adolescents: a nationwide population survey 2017

**DOI:** 10.1186/s12889-020-08916-x

**Published:** 2020-05-20

**Authors:** Yuki Kuwabara, Aya Kinjo, Maya Fujii, Aya Imamoto, Yoneatsu Osaki, Maki Jike, Yuichiro Otsuka, Osamu Itani, Yoshitaka Kaneita, Ruriko Minobe, Hitoshi Maezato, Susumu Higuchi, Hisashi Yoshimoto, Hideyuki Kanda

**Affiliations:** 1 Division of Environmental and Preventive Medicine, Department of Social Medicine, Faculty of Medicine, Tottori University, Nishi-machi 86, Yonago-shi, Tottori, 683-8503 Japan; 2 Department of Public Health, School of Medicine, Nihon University, Itabashi-ku, Tokyo, Japan; 3 National Institute of Alcoholism, Kurihama National Hospital, Yokosuka, Kanagawa Japan; 4 Primary Care and Medical Education, Graduate School of Comprehensive Human Sciences, Majors of Medical Science, University of Tsukuba, Tsukuba, Ibaraki, Japan; 5 Department of Public Health, Okayama University Graduate School of Medicine, Dentistry and Pharmaceutical Sciences, Okayama-shi, Okayama, Japan

**Keywords:** Cigarette smoking, E-cigarettes, Tobacco use, Adolescents, Smoking, Heat-not-burn tobacco, Prevalence

## Abstract

**Background:**

From among the global public health concerns, smoking remains one of the most crucial challenges. Especially for adolescents, the increase in the use of electronic cigarettes is controversial, as its use may lead to established smoking. In Japan, where a unique tobacco regulation system exists, the heat-not-burn tobacco market has been growing. However, the prevalence and association of combustible cigarettes and new tobacco-related products have not yet been closely investigated among Japanese adolescents. This study aimed to clarify the prevalence of smoking among adolescents, including new types of tobacco-related products, and to compare the characteristics of their users.

**Methods:**

The 2017 Lifestyle Survey of Adolescents is a nationally-representative survey collected in Japan. From the national school directory, 98 junior high schools and 86 high schools were randomly sampled throughout Japan. The students completed an anonymous questionnaire at school. We calculated the prevalence of use for each type of tobacco product. Then, the use of a combination of products and the characteristics of different types of products were examined.

**Results:**

In total, 64,152 students from 48 junior high schools and 55 high schools were included the analysis (school response rate = 56%, *M*_age_ = 15.7 years, 53.9% boys). The age-adjusted rate of ever (current) use of electronic cigarettes was 2.1% (0.7%) in junior high school and 3.5% (1.0%) in high school; that of combustible cigarettes was 2.6% (0.6%) in junior high school and 5.1% (1.5%) in high school. The rate of heat-not-burn tobacco use was lower relative to other products: 1.1% (0.5%) in junior high school and 2.2% (0.9%) in high school. An examination of the combined use of the three products identified a high number of dual users. Comparisons between different types of users indicated different backgrounds for combustible cigarette users and new product users.

**Conclusions:**

The prevalence of new tobacco-alternative products is growing in popularity among Japanese adolescents. Dual use is common, and many adolescents use new products only. Moreover, e-cigarettes might attract a broader range of groups to smoking. Continuous monitoring and research are needed to investigate their influence as a possible gateway to tobacco smoking.

## Background

Smoking is a preventable health risk factor that results in numerous diseases and deaths [1, 2]. Smoking control is particularly critical among adolescents, as individuals who use tobacco at a young age are at a considerably higher risk of becoming subsequent smokers [3]. Moreover, tobacco can be a gateway to other types of drug dependence [4]. Therefore, smoking control during adolescence is a crucial public health issue. The National Health Promotion Act has focused on tobacco control, and the prevalence of adolescents who report having smoked combustible cigarettes within last 30 days has continued to decline from 2000 to 2014; 9.4/5.6% (boys/girls) to 1.3/0.6% in junior high school students and 29.9/13.1% to 3.5/1.5% in high school students [5]. However, in recent years, tobacco industries have begun selling new tobacco-related products such as electronic cigarettes (e-cigarettes) and heat-not-burn (HNB) tobacco as alternatives to combustible cigarettes [6].

E-cigarettes use a battery to heat a cartridge containing a liquid, generating steam (i.e. smoke from burning is not generated) [7, 8]. E-cigarettes were first launched in China in 2003, and their consumption has shown a global growth through intense promotion via media geared towards young people, such as YouTube [9, 10]. In smoking surveys worldwide, a rapid rise in the use of e-cigarettes has been seen among adults and adolescents [11–13]. Some of the purported benefits of e-cigarettes are that fewer harmful substances are generated compared to combustible cigarettes [14, 15] and that they can lead to the reduction or cessation of combustible cigarette use. However, there is a concern, particularly for adolescents, that the use of e-cigarettes may cause an increase in established smokers in the future [16]. Moreover, the longitudinal health impact of e-cigarettes has not yet been sufficiently clarified [17]; thus, its use involves potential harm. In Japan, the emergence of e-cigarettes was evaluated by the authorities, and the sale of e-cigarettes containing nicotine is prohibited by the Pharmaceutical Affairs Law of 2010. However, e-cigarettes without nicotine are accessible to adolescents and youth because they are not covered by this law.

HNB tobacco, also known as the ‘I-quit-ordinary-smoking’ (IQOS) system, involves an electronic device that heats tobacco leaves in a stick, and the user inhales the generated aerosol instead of smoke [18]. Tobacco companies in Japan are promoting HNB tobacco as a cigarette that causes less harm to users and bystanders. Philip Morris International (PMI) petitioned the US Food and Drug Administration for approval of HNB tobacco products as a smoking cessation tool, but the application was declined. In 2014, PMI introduced IQOS only in Japan and Italy and was available in 37 countries by August 2018. Japan is the unique country in which HNB tobacco was legally sold nationwide under the Tobacco Industries Act, making it an important market for companies that produce HNB products [19]. The popularity and use of HNB tobacco have increased [20]; in October 2016, Japan comprised 98% of the worldwide IQOS sales [21]. Other HNB products include Japan Tobacco’s Ploom TECH (2016) and British American Tobacco’s Glo product line (2016). Thus, tobacco companies are looking to expand their market offerings [22].

In Japan, where there is a unique market for tobacco-related products, reports on the prevalence of e-cigarette and HNB tobacco use are limited to Internet surveys targeting individuals aged over 18 [23]. As the rapid increase in the popularity of e-cigarettes among adolescents in other countries and of HNB tobacco use in Japan, investigating the prevalence of new products among adolescents is important. Thus, from the 2017 Lifestyle Survey of Adolescents, we obtained data on junior high and high school students’ smoking habits and their use of the new types of tobacco-related products in Japan. Our study aimed to clarify the prevalence of smoking of combustible cigarettes and new tobacco products, as well as the combined use of these products and to compare the background of adolescent users of different types of products.

## Methods

### Study population

This study aimed to evaluate the nationwide prevalence of use of cigarettes and alternative tobacco products. Considering sampling bias, this study involved a cross-sectional random sample survey with single-stage cluster sampling [24], wherein the school was set as the cluster unit. Using the national school directory, junior high schools attended by students aged 12 to 15 and high schools, attended by students aged 15 to 18, throughout Japan were randomly selected, and the survey was distributed to all students in these schools in 2017. A total of 98 of Japan’s 10,325 junior high schools and 86 of the 4907 high schools were sampled. The proportion of private schools was 8.2% of junior high schools and 19.8% of high schools. The survey period was from December 2017 to February 2018.

### Data collection

We asked the school principals for cooperation and sent the survey forms to them for distribution to students through class teachers, who explained to the students that participation was voluntary and that they should answer honestly. The students were given anonymous questionnaires and envelopes, which were completed and sealed by the students, collected by their teachers, and then returned to our research office with the seals intact. This survey was approved by the Ethics Review Committee of Tottori University Faculty of Medicine.

### Measures

The questionnaire survey focused on adolescents’ lifestyle, such as smoking behaviour, alcohol use, and school life (Additional File 1). Referring to the questionnaires used by Centers of Disease Control and Prevention and WHO [25, 26], the questions about smoking included experience with and frequency of combustible cigarette smoking: ‘Have you ever smoked a combustible cigarette including even a single puff?’ and ‘How many days have you smoked combustible cigarettes in the previous 30 days?’. Similar questions were used for new tobacco-alternative products. *Ever users*, *current users*, and *daily users* were defined as those who had smoked even once in the past, had smoked at least once in the past 30 days, and had smoked every day for the past 30 days, respectively. These definitions of frequency were also used for users of e-cigarettes and HNB tobacco.

### Tobacco products

Since we needed to discriminate between combustible cigarettes and new tobacco-alternative products, in the questionnaire, we described a combustible cigarette as ‘a cigarette made from rolled paper and tobacco and smoked with fire’. Due to the number of e-cigarette brands currently for sale, we used the names of the most popular brands in the questionnaire, stating ‘electronic cigarettes include brands such as フレヴォ (FLEVO), エミリ (EMILI), ビタフル(VITAFUL), and ビタシグ(VITASIG)’. The question for HNB tobacco also included product names to avoid any confusion: ‘heat-not-burn tobacco is any product such as アイコス (IQOS), プルームテック (Plume Tech), or グロー (glo)’.

### Data analysis

The age-adjusted prevalence rates were calculated using the number of junior high and high school students nationwide from the School Basic Survey of the Ministry of Education, Science and Technology (2017) as a standard population. Proportions with a 95% confidence interval (95% CI), as presented in the tables, were calculated using a weighting method based on one-stage cluster random sampling [24]. Two proportion *Z*-tests were conducted to compare the prevalence of each product between boys and girls. To observe the associations between the use of the three different types of products, we calculated the prevalence of combined use. Then, the proportions of combined use of products were calculated, which made the comparison of combined use easier. Moreover, the background of ever users of different products was compared in terms of gender, school grade, municipality size, having breakfast, and participating in club activities. IBM SPSS 25.0 was used for all data analyses.

## Results

A total of 56% of 184 schools, including 48 of 98 junior high schools (response rate: 49%) and 55 of 86 high schools (response rate: 64%) took part in the survey. In total, 64,417 questionnaires were returned to the research office. After excluding the questionnaires that were blank, or had invalid/missing gender information or inconsistent responses, 64,152 questionnaires were analysed. The characteristics of the study participants are shown in Table 1. The mean age (standard deviation) of students in junior high school was 13.7 (1.0) years, and 16.7 (0.9) years for high school students. For the gender-ratio, 50.3% of junior high school students and 55.8% of high school students were boys.

**Table 1:** Baseline characteristics of the study participants

	Male		Female		Total	
	*n* = 34,582		*n* = 29,570		*n* = 64,152	
	n	%	n	%	n	%
School grade						
Junior high school (12–15 y/o)						
Grade 7	3740	10.8	3644	12.3	7384	11.5
Grade 8	3687	10.7	3642	12.3	7329	11.4
Grade 9	3702	10.7	3713	12.6	7415	11.6
High school (15–18 y/o)						
Grade 10	7963	23.0	6238	21.1	14,201	22.1
Grade 11	7903	22.9	6309	21.3	14,212	22.2
Grade 12	7470	21.6	5934	20.1	13,404	20.9
Unknown	117	0.3	90	0.3	207	0.3
Municipality size groups						
Large cities	5551	16.1	5968	20.2	11,519	18.0
Cities with populations ≥300,000	10,203	29.5	7288	24.6	17,491	27.3
Cities with populations ≥100,000	11,049	32.0	9339	31.6	20,388	31.8
Cities with populations < 100,000	5995	17.3	5168	17.5	11,163	17.4
Smaller towns and villages	1784	5.2	1807	6.1	3591	5.6
Having breakfast						
Every day	28,070	81.2	25,192	85.2	53,262	83.0
Sometimes	3079	8.9	2600	8.8	5679	8.9
Seldom	2169	6.3	1321	4.5	3490	5.4
Unknown	1264	3.7	457	1.5	1721	2.7
Participating in club activities						
Active	20,106	58.1	16,136	54.6	36,242	56.5
Passive	4667	13.5	4232	14.3	8899	13.9
Not engaging	8477	24.5	8646	29.2	17,123	26.7
Unknown	1332	3.9	556	1.9	1888	2.9

### Rates of cigarette and new tobacco-alternative product use

Broken down by product type and student gender, the age-adjusted rate of students who were ever/current/every day users of cigarettes, e-cigarettes, or HNB tobacco are shown in Table 2.

**Table 2:** Junior high (grades 7–9) and high school (grades 10–12) students’ age-adjusted smoking prevalence rates by gender

	Ever C use		Ever EC use		Ever HNB use	
	%	95% CI	%	95% CI	%	95% CI
Grades 7–9						
Male	3.1	3.0, 3.2	2.4	2.3, 2.5	1.3	1.3, 1.3
Female	2.1**	2.0, 2.2	1.7**	1.6, 1.8	0.9*	0.9, 0.9
Both	2.6	2.5, 2.7	2.1	2.0, 2.2	1.1	1.0, 1.2
Grades 10–12						
Male	6.9	6.6, 7.2	4.9	4.7, 5.1	2.9	2.8, 3.0
Female	3.3**	3.2, 3.4	2.1**	2.1, 2.1	1.4**	1.4, 1.4
Both	5.1	4.8, 5.4	3.5	3.3, 3.7	2.2	2.0, 2.4
	Current C use		Current EC use		Current HNB use	
	%	95% CI	%	95% CI	%	95% CI
Grades 7–9						
Male	0.7	0.7, 0.7	0.8	0.8, 0.8	0.6	0.6, 0.6
Female	0.5	0.5, 0.5	0.5**	0.5, 0.5	0.4	0.4, 0.4
Both	0.6	0.5, 0.7	0.7	0.6, 0.8	0.5	0.5, 0.5
Grades 10–12						
Male	2.0	1.9, 2.1	1.5	1.4, 1.6	1.2	1.1, 1.3
Female	0.9**	0.9, 0.9	0.5**	0.5, 0.5	0.6**	0.6, 0.6
Both	1.5	1.4, 1.6	1.0	0.9, 1.1	0.9	0.8, 1.0
	Daily C use		Daily EC use		Daily HNB use	
	%	95% CI	%	95% CI	%	95% CI
Grades 7–9						
Male	0.2	0.2, 0.2	0.1	0.1, 0.1	0.1	0.1, 0.1
Female	0.1	0.1, 0.1	0.1	0.1, 0.1	0.1	0.1, 0.1
Both	0.1	0.1, 0.1	0.1	0.1, 0.1	0.1	0.0, 0.2
Grades 10–12						
Male	0.7	0.6, 0.8	0.1	0.1, 0.1	0.1	0.1, 0.1
Female	0.2**	0.2, 0.2	0.1	0.1, 0.1	0.0**	0.0, 0.0
Both	0.5	0.4, 0.6	0.1	0.1, 0.1	0.1	0.1, 0.1

*C* combustible cigarette, *EC* electronic cigarette, *HNB* heat-not-burn tobacco, *CI* confidence interval

Two proportion Z-tests were conducted to compare male and female. **P < 0.01, *P < 0.05

The rate of ever users of cigarettes (male/female/both) was 3.1% (95% CI: 3.0, 3.2)/2.1% (95% CI: 2.0, 2.2)/2.6% (95% CI: 2.5, 2.7) for junior high school students, and 6.9% (95% CI: 6.6, 7.2)/3.3% (95% CI: 2.0, 2.2)/5.1% (95% CI: 2.3, 2.9) for high school students. E-cigarette use was slightly lower than cigarette use, at 2.4% (95% CI: 2.3, 2.5)/1.7% (95% CI: 1.6, 1.8)/2.1% (95% CI: 2.0, 2.2) among junior high school students, and 4.9% (95% CI: 4.7, 5.1)/2.1% (95% CI: 2.1, 2.1)/3.5% (95% CI: 3.3, 3.7) among high school students. The rate of HNB tobacco users was relatively lower relative to other products, at 1.3% (95% CI: 1.3, 1.3)/0.9% (95% CI: 0.9, 0.9)/1.1% (95% CI: 1.0, 1.2) among junior high school students, and 2.9% (95% CI: 2.8, 3.0)/1.4% (95% CI: 1.4, 1.4)/2.2% (95% CI: 2.0, 2.4) among high school students. Experience with of all products was significantly higher among adolescent boys than girls.

The rate of current use of the three products (male/female/both) was rare. For cigarettes, the rate was 0.7% (95% CI: 0.7, 0.7)/0.5% (95% CI: 0.5, 0.5)/0.6% (95% CI: 0.5, 0.7) among junior high school students, and 2.0% (95% CI: 1.9, 2.1)/0.9% (95% CI: 0.9, 0.9)/1.5% (95% CI: 1.4, 1.6) among high school students. For e-cigarettes, it was 0.8% (95% CI: 0.8, 0.8)/0.5% (95% CI: 0.5, 0.5)/0.7% (95% CI: 0.6, 0.8) for junior high school students, and 1.5% (95% CI: 1.4, 1.6)/0.5% (95% CI: 0.5, 0.5)/1.0% (95% CI: 0.9, 1.1) for high school students. For HNB tobacco, it was 0.6% (95% CI: 0.6, 0.6)/0.4% (95% CI: 0.3, 0.5)/0.5% (95% CI: 0.5, 0.5) for junior high school students, and 1.2% (95% CI: 1.1, 1.3)/0.6% (95% CI: 0.6, 0.6)/0.9% (95% CI: 0.8, 1.0) for high school students. Among high school students, current use of three products were significantly higher among boys than girls. Significant difference was observed only in e-cigarette use among junior high school students.

The proportion of students who used the products every day was quite low, with the highest prevalence being that of cigarette use among high school students (male/female/both): 0.7% (95% CI: 0.6, 0.8)/0.2% (95% CI: 0.2, 0.2)/0.5% (95% CI: 0.4, 0.6). The age-adjusted rates for the new products were 0.1% or less.

**Fig. 1:**
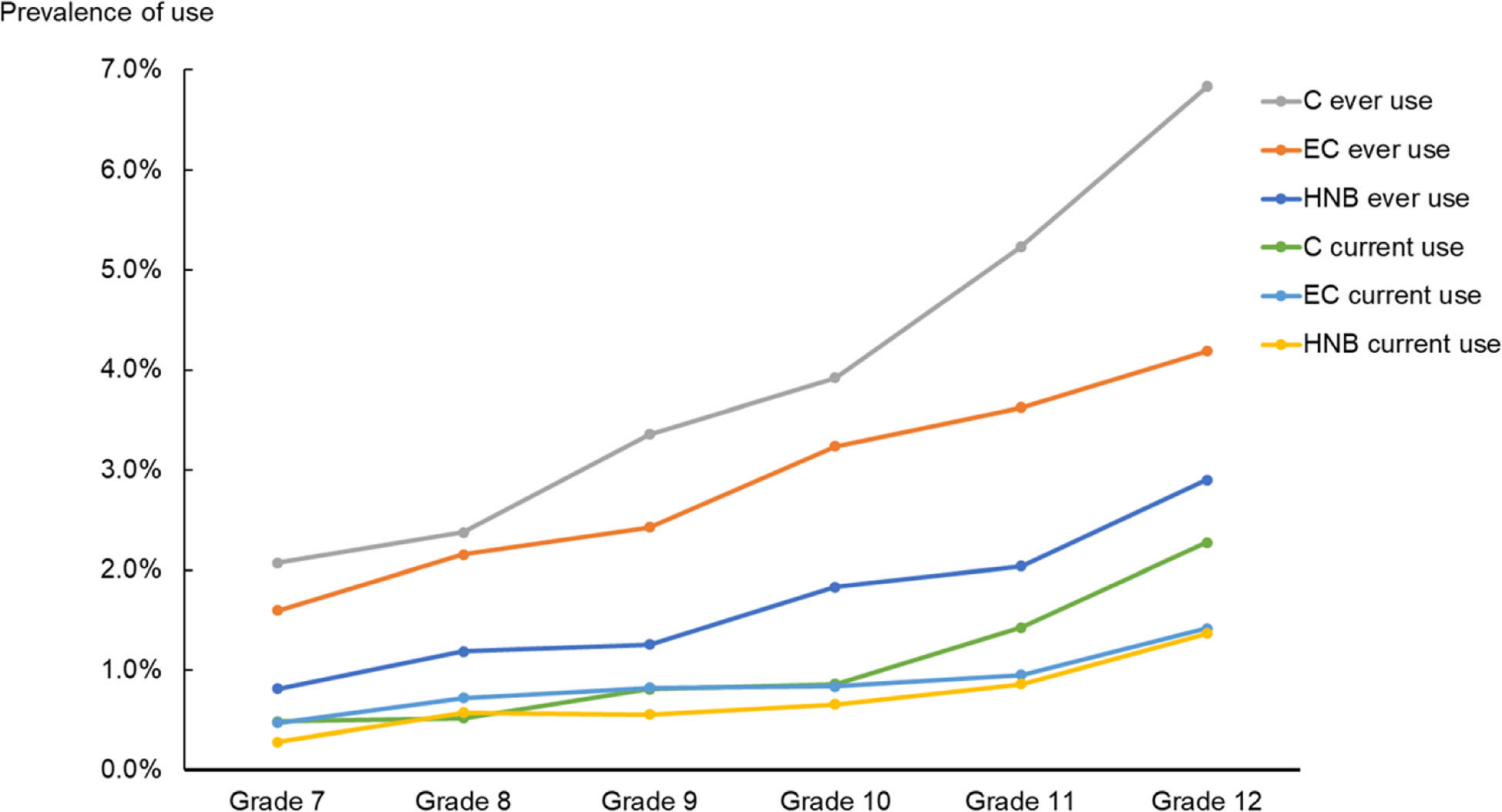
Prevalence of the three tobacco-related products in each grade (both genders). C: combustible cigarette, EC: electronic cigarette, HNB: heat-not-burn tobacco

As shown in Fig. 1, the use of combustible cigarettes was the most prevalent regardless of grade level. Ever use of e-cigarettes followed slightly below that of cigarettes, especially among the younger generation. A divergence was evident between ever use of HNB tobacco and that of the two other types. As for current use, the graphs of the three products overlapped each other in the younger generation, but in grades 11 and 12, combustible cigarette use was significantly higher than the use of the other two products.

### Combined use of any tobacco product

The age-adjusted rates of combined use of tobacco-related products were calculated (Additional File 2), including all possible combinations of combustible cigarettes, e-cigarettes, and HNB tobacco. Across the various patterns, the exclusive users of cigarettes and exclusive users of e-cigarettes were the largest groups.

Moreover, to make comparisons more understandable, the proportions of ever and current users of any product are shown in Table 3. In terms of ever use, the proportion of exclusive users of combustible cigarettes was about 40% of users of any product in both junior high schools and high schools. Meanwhile, around 36% of users of any product used only e-cigarettes and/or HNB tobacco in junior high schools. Among high school users, around 25% of males and 32% of females ever used either one or both new alternative products. Furthermore, among junior high school students who currently used any product, exclusive e-cigarette users were the largest group among all patterns of use. In high school, exclusive combustible cigarette use, 30%, was dominant across the patterns; however, more than 30% of users currently used only new alternative products. It is worth mentioning that dual users who currently used both cigarettes and another type of products exceeded 30% in junior high school as well as in high school.

**Table 3:** Junior high (grades 7–9) and high school (grades 10–12) students’ age-adjusted prevalence of combined smoking by gender

Proportions of students who ever used either								
			Grades 7–9, ever use (%)			Grades 10–12, ever use (%)		
C	EC	HNB	Male	Female	Both	Male	Female	Both
+	–	–	41.1	40.3	40.8	38.4	43.2	39.8
+	+	–	7.8	6.5	7.3	11.6	6.0	10.0
+	–	+	5.6	4.4	5.1	8.1	7.9	8.0
+	+	+	9.6	12.3	10.7	16.7	10.2	14.8
–	+	–	26.3	27.5	26.8	20.0	23.6	21.1
–	–	+	5.0	4.4	4.7	2.7	5.5	3.5
–	+	+	4.6	4.6	4.6	2.4	3.5	2.7
Proportions of students who currently used either								
			Grades 7–9, current use (%)			Grades 10–12, current use (%)		
C	EC	HNB	Male	Female	Both	Male	Female	Both
+	–	–	23.3	20.9	22.4	30.7	29.9	30.5
+	+	–	4.6	3.2	4.1	6.8	6.6	6.8
+	–	+	4.7	9.9	6.6	13.7	17.4	14.6
+	+	+	18.7	28.5	22.4	16.0	14.6	15.7
–	+	–	31.3	28.6	30.3	21.3	16.0	20.0
–	–	+	10.0	7.7	9.1	6.5	9.5	7.3
–	+	+	7.3	1.1	5.0	4.9	6.0	5.1

Proportions excluding those who did not smoke any products

*C* combustible cigarette, *EC* electronic cigarette, *HNB* heat-not-burn tobacco

Additional File 3 shows the comparison of ever users of different products according to gender, school grade, municipality size, custom of having breakfast, and participation in club activities. Across the different users, the proportions of males were higher than those of females. However, gender differences were smaller when comparing e-cigarette users and HNB users with cigarette users. There were upward trends from lower to higher school grades in exclusive cigarette use and cigarette use combined with other products, but the trends were not clear among new alternative products users. Regarding lifestyle, adolescents who ever used new types of products showed acceptable habits. The percentage of individuals who had breakfast every day and participated in club activities was higher among new products users than among cigarette users.

## Discussion

This study is the first in Japan to examine the prevalence of use of combustible cigarettes and new tobacco-alternative products among adolescents. At present, the rate of combustible cigarette use was the highest, followed by e-cigarettes and HNB tobacco use. The rates of use for all three products rose in tandem with increasing school grade. The prevalence of combustible cigarette use was much higher relative to alternative products among high school students, as these alternative products had been introduced into the market more recently. However, ever use of e-cigarettes became close to that of combustible cigarettes among the younger generations. Moreover, the current use of the three products was quite similar, suggesting that new tobacco-alternative products are becoming popular among young users.

Japan is included in the countries with the lowest smoking prevalence according to a World Health Organization report on the global tobacco epidemic [27]. The present study focused on the use of new tobacco-alternative products among Japanese adolescents. However, even considering the increasing prevalence of these new products, the smoking rate has shown a downward trend compared with the respective proportions in time-series data. The current use of e-cigarettes of 0.7% in junior high schools and 1.0% in high schools in Japan is lower than that reported by the 2017 National Youth Tobacco Survey in the US (3.3% in middle school and 11.7% in high school) [28]. The law that regulates the sale of e-cigarettes containing nicotine in Japan might contribute to the lower use of these products. Another explanation may be that HNB tobacco exists as a substitute for e-cigarettes. Even though the current use of new products was low in our study, the use of e-cigarettes has been increasing in several countries. Continuous monitoring of e-cigarette use is thus indispensable.

Moreover, given the large population of e-cigarettes among any products users, it is of significance in assessing whether e-cigarettes used by Japanese adolescents contain nicotine or not. A previous study among Canadian high school students by Hamilton et al. showed that approximately 72% of those adolescents who were ever e-cigarettes users used non-nicotine e-cigarettes, while about 28% used nicotine e-cigarettes [29]. Similarly, a previous study indicated that in Japan, about 30% of ever users of new tobacco products used e-cigarettes containing nicotine [19]. The regulation of e-cigarettes in Canada is quite similar to that in Japan, suggesting that the figures among the Japanese adolescents might be comparable to Hamilton’s results. To note, Tabuchi et al. indicated that about 15% of those who were ever users had used e-cigarettes with unknown nicotine. Misinformation about nicotine content is concerning because nicotine might impact the developing brain of adolescents [29]. Future research should investigate these issues.

The prevalence of HNB tobacco use was lower than that of e-cigarette use; however, HNB tobacco use was still observed among adolescents. To our knowledge, there are no comparable reports about HNB tobacco use in other countries. Tabuchi et al. reported the current use as 3.6% in a 2017 internet survey targeting adults in Japan [23], which is comparable to our results. The prevalence of HNB tobacco use is more similar between adolescents and adults relative to the prevalence of combustible cigarette use. Awareness of and advertisements for HNB tobacco have increased in recent years [22]; a domestic Japanese newspaper reported in 2018 that the sales units of HNB tobacco exceeded 2 million for Glo, 5 million for IQOS, and 4 million for Ploom Tech, and indicated that the tobacco market was accelerating the shift to HNB tobacco [30]. The association between smoking rates and tobacco advertisements has been previously studied, and the increase in the use of e-cigarettes in Western countries has been attributed to media promotion [31]. The current momentum of HNB tobacco in Japan is expected to affect its future use rates. Its prevalence should be monitored, as there is substantial uncertainty regarding the health consequences of HNB tobacco [32, 33].

Gender differences in the prevalence rates were examined in the current study. Boys were more likely to use the tobacco-related products, which is consistent with the results previously reported by the WHO [27] and another study conducted in Japan [19, 23]. Previous research has indicated the importance of investigating gender difference in e-cigarette marketing strategies including innovations in product features (e.g., packaging and device design, appealing flavours), as they can influence gender difference in consumption. The authors also highlighted the necessity of examining gender differences in nicotine use by quantifying the amount of nicotine in the e-cigarettes being used by youth [34]. To our knowledge, no research to date has investigated gender difference in HNB consumption. Future research should examine the context in which gender differences in nicotine use might occur.

This study also examined the various patterns of use of the three products. As for adolescents’ ever use and current use, exclusive cigarette use was dominant in all cases except for the exclusive use of e-cigarettes in junior high school. A considerable proportion of any-product users were ever or current users of new alternative products only. Therefore, it is an important concern whether e-cigarette or HNB tobacco use can lead to established cigarette use in the future. A previous systematic review indicated that the use of e-cigarettes among adolescents was likely to cause subsequent cigarette smoking [16]. Although it is unclear, it is plausible that the use of HNB tobacco use has the same consequences. Future research is required to clarify this issue.

Another concern is ‘dual use’ which refers to the use of both combustible cigarette and at least one new tobacco products. Our results suggest that the proportion of dual users exceeded 30% in junior high school as well as in high school. Although the latest expert consensus indicated that e-cigarette use is much less harmful than smoking combustible cigarettes [17], dual use potentially denotes the addition of unknown harm from e-cigarettes or HNB tobacco to that of smoke from combustible cigarettes [17]. There is controversy over whether dual use of e-cigarettes can assist with smoking cessation or not. A recent systematic review has indicated that e-cigarettes are not likely to lead to smoking cessation [16]. Furthermore, to date, there has been no empirical evidence that has indicated that HNB tobacco products play a role in cessation. Future research should investigate these issues in order to clarify their implications for the overall health impact of e-cigarettes and HNB tobacco in Japan.

Moreover, our results indicated different characteristics in the healthy behaviours between those who were cigarette users and new products users. Previous studies have suggested that smoking is associated with an unhealthy lifestyle [35, 36]. Similarly, an association between health risk behaviour and e-cigarettes use has been reported [37]. However, to our knowledge, the relationship between new products and healthy behaviour among adolescents has not been sufficiently investigated. Dunbar et al. concluded that e-cigarettes use among adolescents is not necessarily associated with greater engagement in health behaviours compared to cigarette use [37]. With regard to HNB tobacco, Lee et al. indicated that physically active adolescents were more likely to use cigarettes as well as new products [38] and theorised that these findings were owing to peer influences from participating social activities. However, our results suggest that new products may be an entrance to smoking for a broad variety of adolescents, who are less likely to begin smoking if using combustible cigarettes alone. Previous studies indicated that new products might entice new groups of consumers with characteristics distinct from those of combustible cigarette smokers [39, 40]. Although the mechanism has not been sufficiently clarified, it could be that new adolescent users may believe that the new products are ‘safe’ [41]. Future studies are needed to examine the association between health behaviours and the type of products used by adolescents. It is also significant to clarify whether young people who are at low-risk of becoming smokers are more attracted by new products.

A strength of the present study is that our large student sample represents the nationwide adolescent population of Japan. In addition, considering our specific tobacco regulation, Japan is a fertile market for tobacco industries. Our study is unique in that it reports on a novel product, HNB tobacco, which is not available in all countries. However, in this survey, the number of schools selected was relatively smaller than in past studies. Additionally, the response rates in junior high schools were low, leading to the use of age-adjusted rates in the tables. This study required strict ethical considerations due to the age groups taking part, which may have contributed to the low response rate. Although we devised questions about e-cigarettes and HNB tobacco, including the trade names of popular products, students still may not have recognised these products correctly and could have confused e-cigarettes and HNB tobacco. It is also difficult to confirm the validity of self-report answers. Continuous monitoring using the same standards and methods may be the only feasible option. Furthermore, this study entailed a cross-sectional analysis; therefore, the temporal relationship of how the smokers’ practices changed as a consequence of the emergence of new tobacco products could not be clarified. Future research should address these limitations.

## Conclusions

According to this nationwide population survey, the prevalence of new tobacco-related products is just below the use of combustible tobacco among Japanese adolescents. Dual use is common, and e-cigarettes or HNB tobacco use represent a considerable proportion of the tobacco-related products used by youth. Findings from background comparison suggest that new tobacco-related products might lure a broader population into smoking. The longitudinal impact of these new products remains unclear; thus, continuous monitoring and further research are necessary to provide guidance for the implementation of enhanced public measures against smoking and the use of new tobacco-related products.

## Supplementary information

**Additional file 1.** The list of questions from the survey questionnaire

**Additional file 2.** Junior high (grades 7–9) and high school (grades 10–12) students’ age-adjusted combined smoking prevalence rates by gender.

**Additional file 3.** Patterns of ever use of tobacco-related products by demographics, lifestyle, and future education intention.

## Data Availability

The datasets used in the current study are available from the corresponding author on reasonable request.

## Abbreviations

E-cigarette: Electronic cigarette
HNB: Heat-not-burn
CI: Confidence interval
WHO: World Health Organization
