# Supplementary material for: Heat-not-burn tobacco, electronic cigarettes, and combustible cigarette use among Japanese adolescents: a nationwide population survey 2017
Source: BMC Public Health. 2020 May 20;20:741. doi: 10.1186/s12889-020-08916-x (PMC7240931; doi:10.1186/s12889-020-08916-x)
Supplement: Supplementary file 1 — Additional file 1. The list of questions from the survey questionnaire [file 12889_2020_8916_MOESM1_ESM.docx]

Additional File 1. The list of questions from the survey questionnaire

※Students were asked to select one option in each question.

**English version**

1. **Demographic data**
2. Are you male or female?

- “Male” or “Female”

1. What is your grade in school?

- “Grade 1”, “Grade 2”, or “Grade 3”

1. How old are you?

- “12”,”13”, “14”, “15”, “16”, “17”, “18” or “more than 19 years old”

1. **Use of cigarettes, e-cigarettes and Heat-Not-Burn tobacco**
2. Have you ever smoked a combustible cigarette including even a single puff? A combustible cigarette made from rolled paper and tobacco and smoked with fire.

- “No, I have not” or “Yes, I have”

1. How many days have you smoked combustible cigarettes in the previous 30 days?

- “0 day”, “1-2 days”, “3-5 days”, “6-9 days”, “10-19 days”, “20-29 days” or “every day”

1. Have you ever smoked an electric cigarette including even a single puff? Electronic cigarettes includeフレヴォ(FLEVO), エミリ (EMILI), ビタフル（VITAFUL), ビタシグ（VITASIG).

- “No, I have not” or “Yes, I have”

1. How many days have you used electric cigarettes in the previous 30 days?

- “0 day”, “1-2 days”, “3-5 days”, “6-9 days”, “10-19 days”, “20-29 days” or “every day”

1. Have you ever used a Heat-Not-Burn tobacco including even a single puff? HNB tobacco was explained by using product names to avoid any confusion; for example, heat-not-burn tobacco includes アイコス（IQOS), プルームテック (Ploom Tech), グロー (glo).

- “No, I have not” or “Yes, I have”

1. How many days have you used Heat-not-Burn tobacco in the previous 30 days?

- “0 day”, “1-2 days”, “3-5 days“, “6-9 days”, “10-19 days”, “20-29 days” or “every day”

1. **Lifestyle behaviours**
2. Do you eat breakfast every day?

- “I have almost every day”, “I have sometimes”, or “I have seldom”

1. Do you participate in club activities?

- “I participate actively”, “I participate but not actively”, or “I do not participate”

**Japanese version**

1. **あなた自身のことについて質問します。**

（１）あなたは男性ですか、女性ですか？あてはまる数字に○をつけてください。

- １．男性　　　　　２．女性

1. あなたの学年は何年生ですか？あてはまる数字に○をつけてください。

- １．１年生　　　　２．２年生　　　　３．３年生

（３）あなたの年齢に○をつけてください。

1．12才　　　　　３．14才　　　　　５．16才　　　　７．18才

２．13才　　　　　４．15才　　　　　６．17才　　　　８．19才以上

1. **ここからはタバコについての質問です。**
2. あなたは、今までに紙巻きタバコ（紙を巻いたぼうの形をしたもので、火をつけて吸う普通のタバコ）を一口でも吸ったことがありますか？

- １．な　い　　　　　　　２．あ　る 

1. この30日間に、何日、紙巻きタバコを吸いましたか？

１．０日　　　３．３～５日　　　　５．10～19日　　　　７．毎日（30日）２．１か２日　４．６～９日　　　　６．20～29日

1. あなたは、今までに電子タバコを一口でも吸ったことがありますか？（電子タバコとは、フレヴォ(FLEVO)、エミリ(EMILI),ビタフル(VITAFUL)、ビタシグ(VITACIG)などの商品のことです）

- １．な　い　　　　　　　２．あ　る

1. この30日間に、何日、電子タバコを吸いましたか？

１．０日　　　３．３～５日　　　　５．10～19日　　　　７．毎日（30日）

２．１か２日　４．６～９日　　　　６．20～29日

1. あなたは、今までに加熱式タバコを一口でも吸ったことがありますか？（加熱式たばことは、アイコス(iQOS)、グロー(glo)、プルームテック(Ploom TECH)のいずれかの商品です）

- １．な　い　　　　　　　２．あ　る

1. この30日間に、何日、加熱式タバコを吸いましたか？

１．０日　　　３．３～５日　　　　５．10～19日　　　　７．毎日（30日）

２．１か２日　４．６～９日　　　　６．20～29日

1. **あなたの生活についてお聞きします。**
2. あなたは、朝食を毎日食べますか？

- １．ほとんど毎日食べている　２．時々食べる　３．ほとんど食べない

1. あなたはクラブ活動に参加していますか？

- １．積極的に参加している ２．積極的でないが参加している

３．参加していない
