# Supplementary material for: Heat-not-burn tobacco, electronic cigarettes, and combustible cigarette use among Japanese adolescents: a nationwide population survey 2017
Source: BMC Public Health. 2020 May 20;20:741. doi: 10.1186/s12889-020-08916-x (PMC7240931; doi:10.1186/s12889-020-08916-x)
Supplement: Supplementary file 2 — Additional file 2. Junior high (grades 7–9) and high school (grades 10–12) students’ age-adjusted combined smoking prevalence rates by gender. [file 12889_2020_8916_MOESM2_ESM.docx]

Additional File 2. Junior high (grades 7–9) and high school (grades 10–12) students’ age-adjusted combined smoking prevalence rates by gender

|  |  |  |  | Grades7–9, ever use | | | | | |  | Grades 10–12, ever use | | | | | |
| --- | --- | --- | --- | --- | --- | --- | --- | --- | --- | --- | --- | --- | --- | --- | --- | --- |
|  |  |  |  | Male | | Female | | Both | |  | Male | | Female | | Both | |
| C | EC | HNB |  | % | 95% CI | % | 95% CI | % | 95% CI |  | % | 95% CI | % | 95% CI | % | 95% CI |
| + | − | − |  | 2.0 | 1.9, 2.1 | 1.3** | 1.3, 1.3 | 1.7 | 1.6,1.8 |  | 3.6 | 3.5, 3.7 | 2.1** | 2.0, 2.2 | 2.9 | 2.8, 3.0 |
| + | + | − |  | 0.4 | 0.4, 0.4 | 0.2* | 0.2, 0.2 | 0.3 | 0.3, 0.3 |  | 1.1 | 1.1, 1.1 | 0.3** | 0.3, 0.3 | 0.7 | 0.6, 0.8 |
| + | − | + |  | 0.3 | 0.3, 0.3 | 0.1* | 0.1, 0.1 | 0.3 | 0.3, 0.3 |  | 0.8 | 0.8, 0.8 | 0.4** | 0.4, 0.4 | 0.6 | 0.5, 0.7 |
| + | + | + |  | 0.5 | 0.5, 0.5 | 0.4 | 0.4, 0.4 | 0.4 | 0.4, 0.4 |  | 1.6 | 1.5, 1.7 | 0.5** | 0.5, 0.5 | 1.1 | 1.0, 1.2 |
| − | + | − |  | 1.3 | 1.3, 1.3 | 0.9* | 0.9, 0.9 | 1.1 | 1.0, 1.2 |  | 1.9 | 1.9, 1.9 | 1.1** | 1.1, 1.1 | 1.6 | 1.6, 1.6 |
| − | − | + |  | 0.2 | 0.2, 0.2 | 0.1 | 0.1, 0.1 | 0.2 | 0.2, 0.2 |  | 0.3 | 0.3, 0.3 | 0.3 | 0.3, 0.3 | 0.3 | 0.3, 0.3 |
| − | + | + |  | 0.2 | 0.2, 0.2 | 0.2 | 0.2, 0.2 | 0.2 | 0.2, 0.2 |  | 0.2 | 0.2, 0.2 | 0.2 | 0.2, 0.2 | 0.2 | 0.2, 0.2 |
| − | − | − |  | 95.2 | 94.4, 96.0 | 96.7 | 95.8, 97.6 | 95.9 | 94.8, 97.0 |  | 92.6 | 91.2, 94.0 | 95.2 | 93.6, 96.8 | 93.6 | 93.1, 94.1 |

|  |  |  |  | Grades 7–9, current use | | | | | |  | Grades 10–12, current use | | | | | |
| --- | --- | --- | --- | --- | --- | --- | --- | --- | --- | --- | --- | --- | --- | --- | --- | --- |
|  |  |  |  | Male | | Female | | Both | |  | Male | | Female | | Both | |
| C | EC | HNB |  | % | 95% CI | % | 95% CI | % | 95% CI |  | % | 95% CI | % | 95% CI | % | 95% CI |
| + | − | − |  | 0.3 | 0.3, 0.3 | 0.2* | 0.2, 0.2 | 0.2 | 0.2, 0.2 |  | 0.9 | 0.8, 1.0 | 0.4** | 0.4, 0.4 | 0.7 | 0.6, 0.8 |
| + | + | − |  | 0.1 | 0.1, 0.1 | 0.0 | 0.0, 0.0 | 0.0 | 0.0, 0.0 |  | 0.2 | 0.2, 0.2 | 0.1** | 0.1, 0.1 | 0.2 | 0.2, 0.2 |
| + | − | + |  | 0.1 | 0.1, 0.1 | 0.1 | 0.1, 0.1 | 0.1 | 0.1, 0.1 |  | 0.4 | 0.4, 0.4 | 0.2** | 0.2, 0.2 | 0.3 | 0.3, 0.3 |
| + | + | + |  | 0.3 | 0.3, 0.3 | 0.2 | 0.2, 0.2 | 0.2 | 0.2, 0.2 |  | 0.5 | 0.5, 0.5 | 0.2** | 0.2, 0.2 | 0.4 | 0.4, 0.4 |
| − | + | − |  | 0.4 | 0.4, 0.4 | 0.2* | 0.2, 0.2 | 0.3 | 0.3, 0.3 |  | 0.7 | 0.7, 0.7 | 0.2** | 0.2, 0.2 | 0.5 | 0.5, 0.5 |
| − | − | + |  | 0.1 | 0.1, 0.1 | 0.1 | 0.1, 0.1 | 0.1 | 0.1, 0.1 |  | 0.2 | 0.2, 0.2 | 0.1 | 0.1, 0.1 | 0.2 | 0.2, 0.2 |
| − | + | + |  | 0.3 | 0.3, 0.3 | 0.2** | 0.2, 0.2 | 0.2 | 0.2, 0.2 |  | 0.5 | 0.5, 0.5 | 0.2 | 0.2, 0.2 | 0.4 | 0.4, 0.4 |
| − | − | − |  | 98.6 | 97.8, 99.4 | 99.2 | 98.3, 100 .0 | 98.9 | 97.8, 100.0 |  | 98.9 | 97.3, 100.0 | 98.7 | 97.1, 100.0 | 98.8 | 98.4, 99.2 |
| C: combustible cigarette, EC: electronic cigarette, HNB: heat-not-burn tobacco, CI: confidence interval. | | | | | | | | | | | | | | | | |
| Two proportion Z-tests were conducted to compare male and female. **P<0.01, *P<0.05. | | | | | | | | | | | | | | | | |
