# Supplementary material for: Heat-not-burn tobacco, electronic cigarettes, and combustible cigarette use among Japanese adolescents: a nationwide population survey 2017
Source: BMC Public Health. 2020 May 20;20:741. doi: 10.1186/s12889-020-08916-x (PMC7240931; doi:10.1186/s12889-020-08916-x)
Supplement: Supplementary file 3 — Additional file 3. Patterns of ever use of tobacco-related products by demographics, lifestyle, and future education intention. [file 12889_2020_8916_MOESM3_ESM.docx]

Additional File 3. Patterns of ever use of tobacco-related products by demographics, lifestyle, and future education intention

|  |  | C users | | | Exclusive C users | |  | Exclusive EC users | | Exclusive HNB users | | | |
| --- | --- | --- | --- | --- | --- | --- | --- | --- | --- | --- | --- | --- | --- |
|  |  | n=2,808 | |  | n=1,593 | |  | n=890 | | | n=150 | | |
|  |  | n | % |  | n | % |  | n | % |  | | n | % |
| Gender | |  |  |  |  |  |  |  |  |  | |  |  |
|  | Male | 1967 | 70.0 |  | 1055 | 66.2 |  | 576 | 64.7 |  | | 85 | 56.7 |
|  | Female | 841 | 30.0 |  | 538 | 33.8 |  | 314 | 35.3 |  | | 65 | 43.3 |
| School grade | |  |  |  |  |  |  |  |  |  | |  |  |
|  | Junior high school |  |  |  |  |  |  |  |  |  | |  |  |
|  | Grade 7 | 153 | 5.4 |  | 103 | 6.5 |  | 67 | 7.5 |  | | 15 | 10.0 |
|  | Grade 8 | 174 | 6.2 |  | 109 | 6.8 |  | 84 | 9.4 |  | | 15 | 10.0 |
|  | Grade 9 | 249 | 8.9 |  | 156 | 9.8 |  | 91 | 10.2 |  | | 13 | 8.7 |
|  | High school |  |  |  |  |  |  |  |  |  | |  |  |
|  | Grade 10 | 557 | 19.8 |  | 321 | 20.2 |  | 236 | 26.5 |  | | 47 | 31.3 |
|  | Grade 11 | 744 | 26.5 |  | 415 | 26.1 |  | 217 | 24.4 |  | | 32 | 21.3 |
|  | Grade 12 | 916 | 32.6 |  | 480 | 30.1 |  | 192 | 21.6 |  | | 28 | 18.7 |
|  | Unknown | 15 | 0.5 |  | 9 | 0.6 |  | 3 | 0.3 |  | | 0 | 0.0 |
| Municipality size groups | |  |  |  |  |  |  |  |  |  | |  |  |
|  | Large cities | 508 | 18.1 |  | 296 | 18.6 |  | 146 | 16.4 |  | | 23 | 15.3 |
|  | Cities with populations ≥300,000 | 794 | 28.3 |  | 443 | 27.8 |  | 260 | 29.2 |  | | 39 | 26.0 |
|  | Cities with populations ≥100,000 | 985 | 35.1 |  | 520 | 32.6 |  | 289 | 32.5 |  | | 57 | 38.0 |
|  | Cities with populations <100,000 | 339 | 12.1 |  | 230 | 14.4 |  | 143 | 16.1 |  | | 19 | 12.7 |
|  | Smaller towns and villages | 182 | 6.5 |  | 104 | 6.5 |  | 52 | 5.8 |  | | 12 | 8.0 |
| Having breakfast | |  |  |  |  |  |  |  |  |  | |  |  |
|  | Every day | 1809 | 64.4 |  | 1109 | 69.6 |  | 663 | 74.5 |  | | 111 | 74.0 |
|  | Sometimes | 477 | 17.0 |  | 233 | 14.6 |  | 109 | 12.2 |  | | 20 | 13.3 |
|  | Seldom | 408 | 14.5 |  | 196 | 12.3 |  | 77 | 8.7 |  | | 14 | 9.3 |
|  | Unknown | 114 | 4.1 |  | 55 | 3.5 |  | 41 | 4.6 |  | | 5 | 3.3 |
| Participating in club activities | |  |  |  |  |  |  |  |  |  | |  |  |
|  | Active | 1139 | 40.6 |  | 719 | 45.1 |  | 449 | 50.4 |  | | 67 | 44.7 |
|  | Passive | 411 | 14.6 |  | 219 | 13.7 |  | 132 | 14.8 |  | | 30 | 20.0 |
|  | Not engaging | 1136 | 40.5 |  | 596 | 37.4 |  | 268 | 30.1 |  | | 46 | 30.7 |
|  | Unknown | 122 | 4.3 |  | 59 | 3.7 |  | 41 | 4.6 |  | | 7 | 4.7 |
| C: combustible cigarette, EC: electronic cigarette, HNB: heat-not-burn tobacco. | | | | | | |  |  |  |  | |  |  |
